# Selecting interventions to improve patient-relevant outcomes in health care for aortic valve disease – the Intervention Selection Toolbox

**DOI:** 10.1186/s12913-020-05090-z

**Published:** 2020-03-19

**Authors:** Nina Zipfel, A. Stef Groenewoud, Benno J. W. M. Rensing, Edgar J. Daeter, Lea M. Dijksman, Jan-Henk E. Dambrink, Philip J. van der Wees, Gert P. Westert, Paul B. van der Nat

**Affiliations:** 1 Department of Value-based Healthcare, St. Antonius Hospital, P.O. Box 2500, 3430 EM Nieuwegein, the Netherlands; 2 Radboud university medical center, Radboud Institute for Health Sciences, Scientific Center for Quality of Healthcare (IQ healthcare), P.O. Box 9101, 6500 HB Nijmegen, the Netherlands; 3 Department of Cardiology, St. Antonius Hospital, P.O. Box 2500, 3430 EM Nieuwegein, the Netherlands; 4 Department of Cardiothoracic Surgery, St. Antonius Hospital, P.O. Box 2500, 3430 EM Nieuwegein, the Netherlands; 5 Department of Cardiology, Isala klinieken, P.O. Box 10400, 8000 GK Zwolle, the Netherlands

**Keywords:** Quality improvement, Patient outcomes, Quality management, Value-based healthcare

## Abstract

**Background:**

Measuring and improving outcomes is a central element of value-based health care. However, selecting improvement interventions based on outcome measures is complex and tools to support the selection process are lacking. The goal was to present strategies for the systematic identification and selection of improvement interventions applied to the case of aortic valve disease and to combine various methods of process and outcome assessment into one integrated approach for quality improvement.

**Methods:**

For this case study a concept-driven mixed-method approach was applied for the identification of improvement intervention clusters including: (1) benchmarking outcomes, (2) data exploration, (3) care delivery process analysis, and (4) monitoring of ongoing improvements. The main outcome measures were long-term survival and 30-day mortality. For the selection of an improvement intervention, the causal relations between the potential improvement interventions and outcome measures were quantified followed by a team selection based on consensus from a multidisciplinary team of professionals.

**Results:**

The study resulted in a toolbox: the Intervention Selection Toolbox (IST). The toolbox comprises two phases: (a) identifying potential for improvement, and (b) selecting an effective intervention from the four clusters expected to lead to the desired improvement in outcomes. The improvements identified for the case of aortic valve disease with impact on long-term survival in the context of the studied hospital in 2015 include: anticoagulation policy, increased attention to nutritional status of patients and determining frailty of patients before the treatment decision.

**Conclusions:**

Identifying potential for improvement and carefully selecting improvement interventions based on (clinical) outcome data demands a multifaceted approach. Our toolbox integrates both care delivery process analyses and outcome analyses. The toolbox is recommended for use in hospital care for the selection of high-impact improvement interventions.

## Background

The importance of improving outcomes in health care has widely been recognized [1–5], while the improvement of quality in health care is a science in itself [6]. Closely linked is the science of outcome research, which has been accepted in research as a “foundation of knowledge about what constitutes ideal care and what gaps exist between ideal and actual care” [7]. Measuring and improving outcomes is a central element of value-based health care (VBHC) [8]. However, selecting improvement interventions based on outcome measures is complex and tools to support the selection process are lacking. Improvement interventions are interventions or tools that change processes leading to improved quality of care [9, 10]. For the purpose of this study, improvement interventions may concern any deliberate action aimed at achieving positive change in outcomes through structure and/or process interventions.

Value-based health care aims at achieving higher value for patients relative to the costs [11]. In order to achieve a value-based system, care delivery should be organized around health conditions. The care delivery value chain (CDVC) describes activities that add value for patients [12] and can be used to analyze processes to maximize this value for patients. In the CDVC, value of a single activity can only be understood by considering the full cycle of care and thus the relation to other care delivery activities [12].

In the literature several quality improvement models are presented [5, 13–15]. For example, the “Implementation of Change Model” for achieving change in a systematic manner [5]. They identified a seven-step plan to successfully implement change for improving the quality of health care delivery [16]. However, this model lacks a focus on outcome measures as a basis for the identification of improvement initiatives. Furthermore, the literature suggests “a clinical value compass” as a method to select an improvement intervention, which measures on the following four domains: (1) functional status, risk status, and well-being, (2) costs, (3) satisfaction with health care and perceived benefit, and (4) clinical outcomes [13]. This method lacks a step for identifying improvement potential. Another possible method for the identification of an improvement intervention could be the plan-do-study-act (PDSA) model [14]. The PDSA model focuses on processes of care delivery in order to achieve improvement and change. However, it does not offer clear tools on how to identify and select a focus for improvement. A different approach for improving quality of care is benchmarking. Benchmarking is the process of identifying so-called “best practices”, which are the highest excellence standards [17]. Benchmarking means identifying good practices as a result of comparisons with other organizations that lead to better patient-relevant outcomes [18]. Benchmarking can take place on different levels, for example as performance comparisons, process comparisons, or strategic comparisons [17]. Another method described to change processes of care in order to improve the quality of care is “Lean thinking”, which puts process evaluation central [15], and focuses on reducing waste and synchronizing work flows to combat and manage variability in work flow [15]. Six Sigma has been introduced along with Lean in order to improve the organizational structure through improvement projects while making use of the several steps [15]. It lacks outcome measures and focuses merely on structure indicators. All these models use different approaches or cycles for continuous quality improvement. However, all of them lack an explicit focus on patient-relevant outcome measures when designing an improvement intervention.

This paper integrates the identification and selection of improvement interventions, the focus on patient-relevant outcomes, and underlying care delivery processes into a single coherent approach. The primary aim is to develop a toolbox for selecting improvement interventions that positively influence health outcomes in the right direction. The secondary aim is to apply this toolbox to aortic valve disease. For this aim we used outcome data from the clinical outcome registry of the Dutch national initiative Measurably Better (MB). MB is an initiative in the Netherlands that aims to improve quality and transparency of care for patients with heart diseases using patient-relevant outcome measures [19]. In 2017, MB merged with the national registries for cardiology and thoracic surgery forming the Netherlands Heart Registry [20]. MB offers the infrastructure to construct a case for the development and application of a toolbox.

The overall goal is to provide health care professionals with a tool that fills the existing gap between measuring and improving patient-relevant health outcomes.

## Methods

### Case study setting

We chose a single case-study design. We then purposefully selected a nested single case in order to understand strategies on how to identify and select improvement initiatives based on the VBHC concept [21]. MB was selected, because it offered the needed infrastructure. The setting of the case study was a Dutch non-academic teaching hospital with a high volume cardiac intervention center. The focus of the case is aortic valve disease with a specific focus on two treatment modalities: Surgical Aortic Valve Replacement (SAVR) and Transcatheter Aortic Valve Replacement (TAVR). The analysis was conducted by means of chronological description. A non-medical scientific research declaration was obtained from the Medical Research Ethics Committees United (MEC-U) of the St. Antonius Hospital with the following reference number: W15.006.

### Methodological approach: concept-driven mixed-method approach

**Fig. 1:**
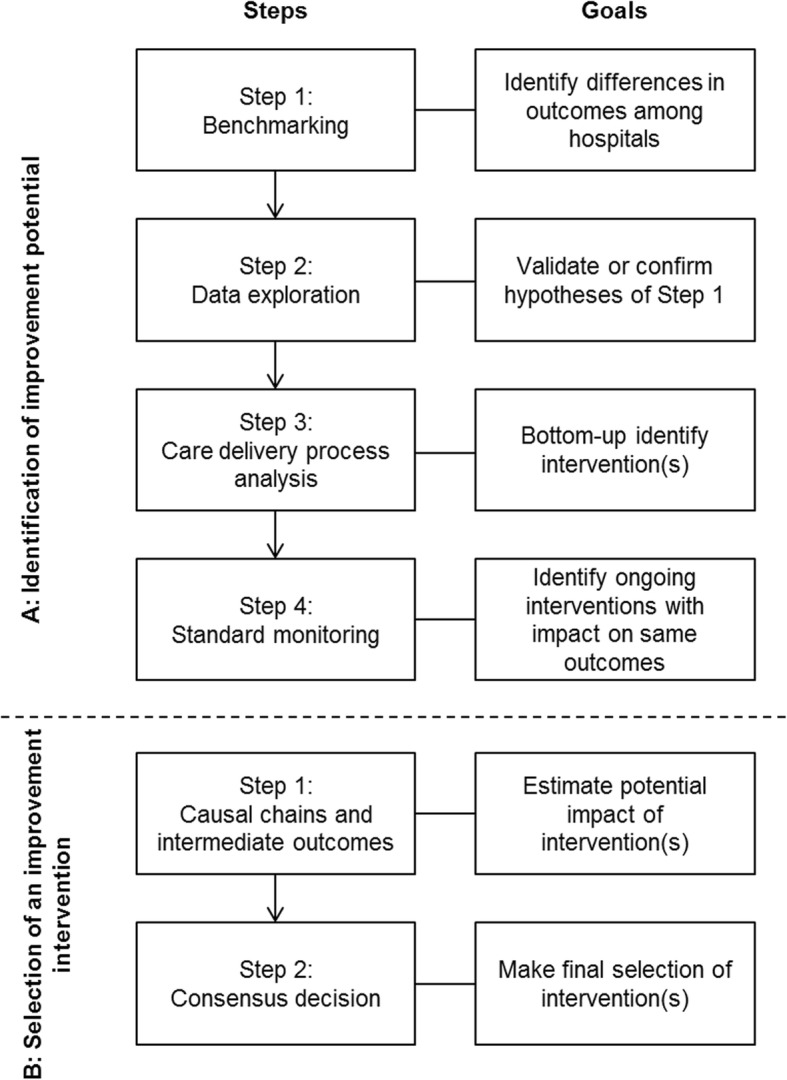
Flowchart of methodological mixed-method approach. Flowchart of methodological mixed-method approach for (**a**) the identification of improvement potential and (**b**) selection of an improvement intervention describing the goals of each step

This paper describes a strategy including four steps for (A) the identification of improvement potential, and two steps for (B) the selection of improvement interventions*.* Figure 1 presents a flow chart of all methodological steps and their goals. A multidisciplinary team, led by a project team consisting of researchers (*N* = 2), was involved to collect expert opinions from all stakeholders in the care delivery process for aortic valve disease. The multidisciplinary team was formed in June 2015 and consisted of cardiologists (*N* = 2), cardiothoracic surgeons (*N* = 2), nurses (*N* = 2), anaesthesiologists (*N* = 2), a data manager (*N* = 1) and researchers (*N* = 2) of the St. Antonius Hospital in the Netherlands. Verbal consent to participate in the multidisciplinary team was obtained before participation.

A: Identification of improvement potential

The identification of potential for improvement consisted of four steps: benchmarking, data exploration, care delivery process analysis and monitoring. The four steps are described chronologically.

- Step 1: Benchmarking

In the first step, called “benchmarking”, we conducted a systematic analysis to identify meaningful differences in patient-relevant outcomes among hospitals. In general, benchmarking includes the following steps: identification of outcomes to be benchmarked, establish organization to benchmark with, collect data, analyse for differences, determine future trends and reveal results. For our analyses we used the annual report of MB, including outcome data of 19 Dutch heart centers [22]. The outcome measures that were used are long-term survival, 120-day mortality, 30-day mortality (only TAVR), Quality of Life, cerebrovascular accident (CVA), deep sternal wound infection (only SAVR), implantation of a new permanent pacemaker, vascular complications (only TAVR) and freedom of valve re-intervention [20, 22, 23]. For detailed definitions see Additional file 1.

The multidisciplinary team discussed the outcome measures indicated by the measurements to have a below average performance or a negative (absolute or relative) trend over time of the primary hospital. The team decided whether differences observed in outcomes were clinically relevant and subsequently formulated hypotheses for the probable causes of these differences.

- Step 2: Data exploration

Data exploration is a method to understand data and their characteristics. For this step, we performed data analyses to validate or confirm the hypotheses of Step 1. In addition, further analyses were performed to identify subgroups of the total patient population with higher risks of negative outcomes. To be able to perform these analyses, five hospitals from MB provided patient-level data over the period from 2010 to 2014 [20]. We tested these hypotheses with univariable and multivariable logistic regression and applied these methods to identify significant predictors of 30-day mortality. The goal was to explain possible causes of differences in long-term mortality by giving more insights into differences between the 30-day mortality of the primary hospital and other MB hospitals. We conducted an additional Cox-regression analysis for insights into the 30-day survival. All analyses were conducted with IBM SPSS statistics 22 [24]. We further complemented this step with literature research in order to find possible improvement interventions fitting the risk groups identified. Literature was searched based on search terms resulting from the data analyses including risks, patient-relevant outcomes, processes and mortality.

- Step 3: Care delivery process analysis

In the third step we conducted a CDVC analysis for aortic valve disease (Additional file 2) [12]. In this analysis, the care process was laid out describing all processes for the full cycle of care of a disease. Following, the care processes were prioritized by the multidisciplinary team. The aim of this step was two-fold: to identify specific interventions that could possibly improve the patient-relevant outcomes and to gather additional bottom-up identification of improvement interventions. The multidisciplinary team used a scoring tool based on the CDVC framework to score each process component per treatment based on the following criteria: (1) impact on patient-relevant outcomes, (2) room for improvement, and (3) feasibility to improve. For every potential improvement intervention the multidisciplinary team members were asked to link it to one of the outcome measures used by MB (Additional file 3). After a compilation and evaluation of the ranking, we organized a second expert session to discuss and present results, with the aim to identify possible improvement interventions. The result was a list of interventions.

- Step 4: Standard monitoring

A fourth step was used to monitor and integrate ongoing improvements that could impact patient-relevant outcomes. Monitoring ongoing improvement could include a list of improvement interventions with their associated processes and/or outcomes. This monitoring step is needed to identify potential ongoing improvement interventions with impact on the same outcome measures as identified in Step 1 and 2. What is also needed is an overview of ongoing improvement interventions to be able to judge the added value of the improvement interventions resulting from Step 1–3. We regularly updated the standard monitoring whenever new improvement interventions were started up at the primary hospital. A list of ongoing improvement interventions linked to outcome measures resulted from this step.

B: Selection of an improvement intervention

After the identification, we needed to select an improvement intervention, which required two steps.

- Step 1: Causal chains and intermediate outcomes

**Fig. 2:**
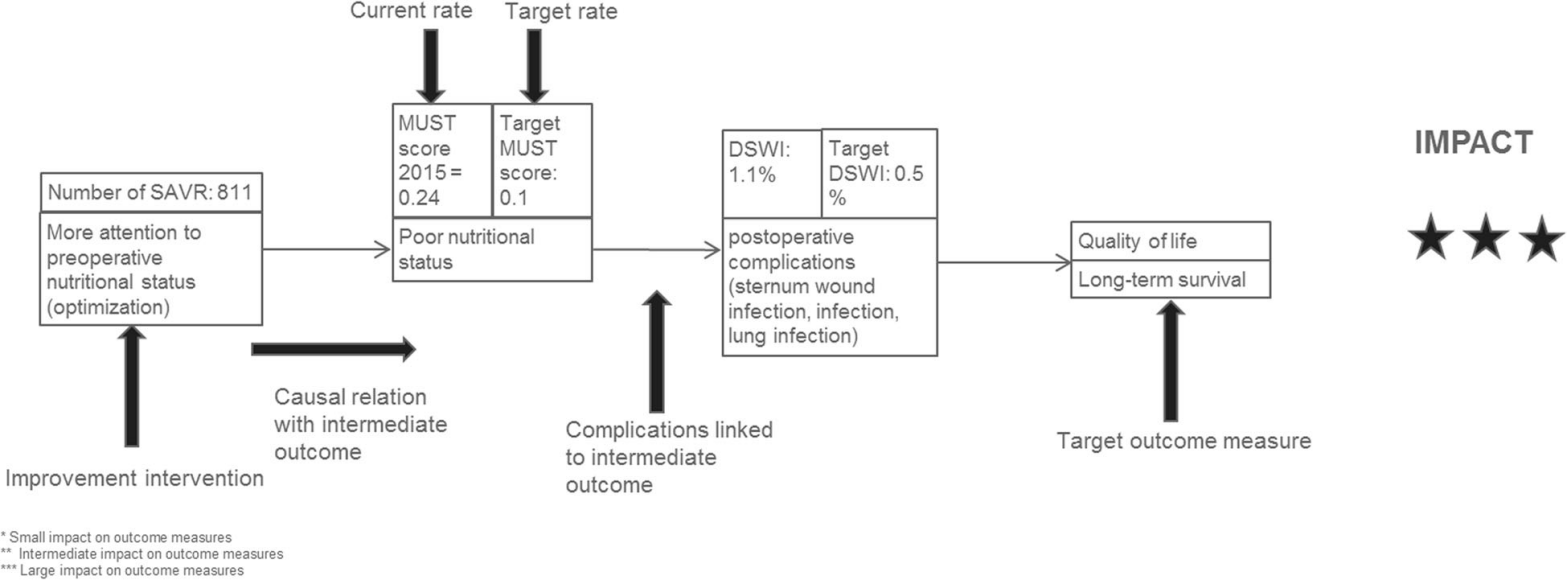
Example of a causal chain. MUST is the Malnutrition Universal Screening Tool. DSWI is deep sternum wound infection. One star indicates a small impact on outcome measures. Two stars indicate a slightly bigger (intermediate) impact on outcome measures. Three starts represent a large impact on outcome measures

The goal of the first step was to analyze the impact of potential improvement interventions on patient-relevant outcomes. To estimate the potential impact of the improvement interventions on the outcome measures, we developed and performed a causal chain analysis (Fig. 2). A causal chain is the path from improvement intervention to outcome measure. In between the intervention and a patient-relevant outcome are intermediate outcomes, which are outcomes that are impacted more directly by the intervention. Intermediate outcomes were relevant for monitoring the impact of an improvement intervention. They also allow for proving an effect when the impact on the outcome measures would be too small to measure statistically significant impact. The results of A formed the basis for this step. Two researchers and a cardiologist ranked the results according to relevance. Relevance was scored on a three-star scale from limited to high impact with the following criteria which were added to an overall score: (a) impact on the outcome measure, (b) technical and practical feasibility, and (c) feasibility in terms of costs. The aim of this ranking was to narrow down a pre-selection to offer a sharper scope of the possible improvement interventions.

- Step 2: Consensus decision

In the second step we used an adjusted Delphi method to make the final selection of the improvement intervention(s). The multidisciplinary team was asked to score the improvement interventions once with the information on the causal chains according to the impact on patient-relevant outcomes during a team meeting. The multidisciplinary team was given the chance to revise their choice at the end of the first round of prioritization. The final decision was made at the end of the meeting and follow-up meetings were organized to further design implementation of the intervention.

## Results

A: Identification of improvement potential

- Step 1: Benchmarking

Benchmarking resulted in one outcome measure for both SAVR and TAVR: long-term survival. We observed a difference in long-term survival between the primary hospital and the other hospitals in the benchmark [22]. This result led to formulating the following hypotheses for follow-up data analyses with the goal of explaining the differences:

1. There are no differences in survival within 30 days for SAVR.
2. Differences in long-term survival for TAVR can be attributed to a number of explanatory variables and do not persist in 30-day mortality.

- Step 2: Data exploration

**Fig. 3:**
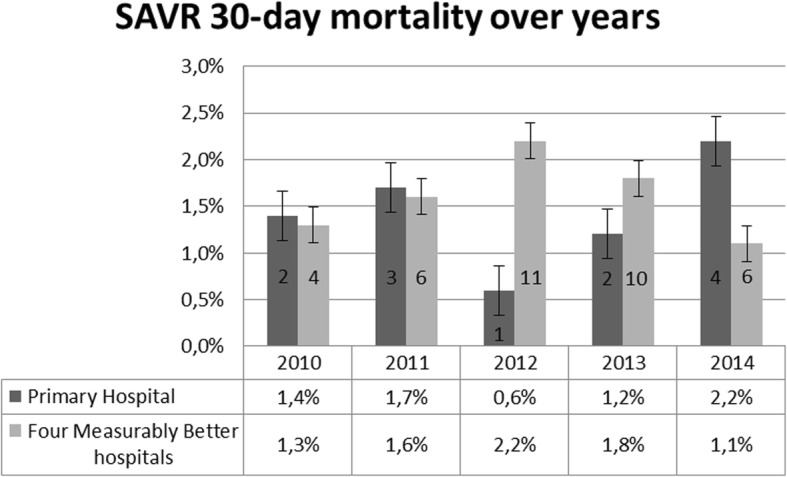
SAVR 30-day mortality for the primary hospital and four MB hospitals over time. Measurably Better data report 2015. Including the number of cases occurred per year for the primary hospital and four Measurably Better hospitals

**Fig. 4:**
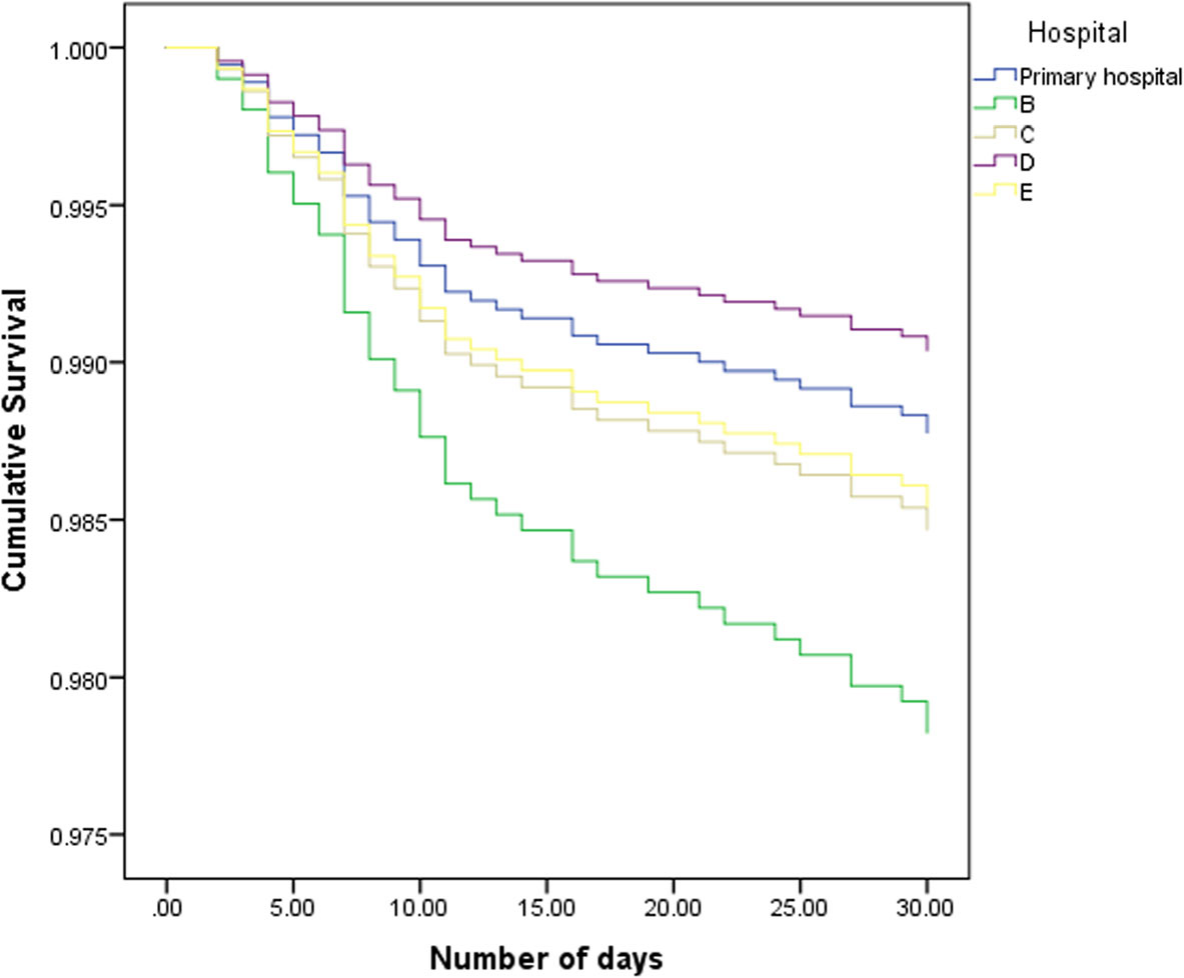
Cox-regression survival curves within 30 days after procedure. Primary hospital compared to four hospitals corrected for EuroSCORE. Procedural mortality was excluded for this analysis. Analysis starts at 1 day post-procedure. Hospital B (*N* = 318) (HR 1.79, 95% CI 0.7–4.57, *p* = 0.224), hospital C (*N* = 359) (HR 1.26, 95% CI 0.46–3.46, *p* = 0.661), hospital D (*N* = 947) (HR 0.79, 95% CI 0.33–1.9, *p* = 0.592), hospital E (*N* = 618) (HR 1.2, 95% CI 0.5–2.88, *p* = 0.694) did not differ significantly from the primary hospital (*N* = 822) in survival within 30 days after procedure

We tested the hypotheses, to explore whether unfavorable results in long-term survival occurred due to factors that can be attributed to the operation and operating technique (Additional file 1). We conducted the SAVR analysis for the primary hospital and compared it to available data from four MB hospitals; we did not correct it for other explanatory variables. The analysis of the 30-day mortality of the SAVR treatment is shown in Fig. 3. The insights into the 30-day mortality for SAVR was not considered sufficient to identify whether differences in long-term survival can be attributed to factors linked to the operation. Therefore, we conducted an additional Cox-regression to identify differences in survival within 30 days after the procedure. These insights would help identify a focus for improvement; improvement around the procedure or improvement with impact on long-term survival. We excluded procedural mortality for this analysis, because the focus was not on mortality during the operation, but post-surgery. Moreover, 23 cases had missing values and were for that reason excluded from the analysis. The primary hospital did not differ significantly in survival within 30 days after the procedure from the other participating hospital (hospital B: HR 1.79, 95% CI 0.7–4.57, *p* = 0.224; hospital C: HR 1.26, 95% CI 0.46–3.46, *p* = 0.661; hospital D: HR 0.79, 95% CI 0.33–1.9, *p* = 0.592; hospital E: HR 1.19, 95% CI 0.5–2.88, *p* = 0.694) (Fig. 4). Both the crude analysis and the Cox-regression gave valuable insights into crude differences in hospitals and showed that potential to improve could possibly be achieved by QI targeting long-term survival instead of 30-day mortality and procedural improvements. Furthermore, the hypothesis was tested whether 30-day mortality can be explained by valve type at the primary hospital. The result of the logistic regression model for SAVR was not statistically significant (Table 1).

**Table 1:** Univariable and multivariable logistic regression results predicting 30-day mortality

Treatment	Predictor	Category	Univariable			Multivariable		
			OR	(95% CI)	*p* value	OR	(95% CI)	*p* value
SAVR (*N* = 3760)	Valve type	Bio prosthetic valve	1.0					
		Mechanical valve	1.5	(0.32–6.88)	.607			
TAVR (*N* = 1929)	Access route	Direct aortic	1.0			1.0		
		Transfemoral	0.5	(0.28–0.80)	0.006	0.4	(0.19–0.75)	0.005
		Transapical	1.4	(0.83–2.47)	0.196	1.4	(0.65–2.87)	0.417
	Vascular complication		2.5	(1.66–3.70)	< 0.001	2.9	(1.92–4.63)	< 0.001
	Valve re-intervention		0.8	(0.19–3.66)	0.819			
	Previous heart operation		0.9	(0.67–1.45)	0.932			
	Previous CVA^a^		1.4	(0.85–2.14)	0.203			
	Previous mitral valve stenosis		0.6	(0.4–0.96)	0.033	1.4	(0.84–2.22)	0.213
	Hospital^b^	Primary hospital	1.0			1.0		
		A	0.7	(0.46–1.19)	0.214	1.0	(0.56–1.80)	0.993
		B	0.7	(0.43–0.98)	0.041	0.9	(0.54–1.47)	0.658
		C	1.1	(0.7–1.71)	0.691	0.2	(0.09–0.57)	0.002
		D	0.4	(0.21–0.76)	0.005	0.2	(0.06–0.68)	0.010
		E	0.4	(0.16–1.05)	0.063	0.09	(0.01–0.70)	0.021
	Urgency^c^	Elective	1					
		Urgent	0.8	(0.48–1.33)	0.390			
	Severe left ventricular dysfunction	> 50%	0.6	(0.21–1.77)	0.363			
		< 50%	1.0	(0.33–2.77)	0.935			
	Age		1.0	(0.98–1.06)	0.427			
	Renal dysfunction		1.6	(1.13–2.27)	0.008	1.9	(1.27–2.82)	0.002

^a^CVA cerebrovascular accident

^b^Analysis for Hospital was conducted relative to the primary hospital. Measurably Better data 2015

^c^Urgency: for urgent operations, no emergency and rescue operations

For TAVR we conducted univariable logistic regression analysis (Table 1). Due to the small amount of cases for the subclavian access route we added cases to the transapical category, and transaxillary cases to the direct aortic category. For this analysis we also excluded emergency and rescue cases due to the small amount of cases (*N* = 3). For the 30-day mortality four missing values were identified and excluded from the analysis. The only variables found to be independent predictors for 30-day mortality were transfemoral access route (OR 0.5, 95% CI 0.28–0.80, *p* = 0.006), vascular complication (OR 2.5, 95% CI 1.66–3.70, *p* < 0.001), previous mitral valve stenosis (OR 0.6, 96% CI 0.4–.096, *p* = 0.033), hospital B (OR 0.7, 95% CI 0.43–0.98, *p* = 0.041), hospital D (OR 0.4, 95% CI 0.21–0.76, *p* = 0.005) and renal dysfunction (OR 1.6, 95% CI 1.13–2.27, *p* = 0.008) (Table 1). There was no difference in outcome between a logistic regression model that included variables with a *p* value < 0.1 in the univariable analysis and a model that included variables with a *p* value < 0.05. The Hosmer-Lemeshow test showed a goodness of fit (χ^2^ = 13.28, *p* = 0.066). The results provided us with valuable insights into predictors and hospitals associated with 30-day mortality, which led to contact with hospitals. The identification of significant predictors also helped to set the focus for higher risk groups of patients.

- Step 3: Care delivery process analysis

Step 3 resulted in total in 40 potential improvement initiatives (Table 2). Those potential improvements were the result of the focus set on higher risk groups of patients in step 2 and the contact with other hospitals. We identified eighteen improvement interventions for SAVR. The care delivery process analysis resulted in several interventions that aim to improve awareness toward care for older patients. In the TAVR care delivery process analysis we identified 22 improvement initiatives.

- Step 4: Standard monitoring

**Table 2:** Results care delivery process analysis

Treatment	Process Phase	Potential improvement intervention	Impact on outcome
SAVR	Monitoring and preventing	Identify high-risk patients by measuring a Frailty Score	Mortality, Quality of Life
		Organize a specific pre-operative screening for older patients	None*
	Diagnosing	Introduce a frailty protocol	Quality of Life, mortality
		Discuss older patients in a multidisciplinary team	Quality of Life, Mortality
		Introduce a checklist for uniform imaging	Quality of Life, Mortality
		Screen abdominal vascular disease	Mortality
		Screen for long-vein narrowing	Mortality
	Preparing	Adjust the anticoagulation protocol	Mortality
	Intervening	Standardize with a protocol for the blood or crystalloid cardioplegia	Mortality
		Use of MECC^a^ and improve experience of the operation team	Mortality
		Implant the long-term pacemaker as fast as possible after operation	Mortality
	Recovery/Rehab	Conduct an echocardiography only with indication	Quality of Life
		Improve nightly supervision at the ICU^b^ (cultural change)	Mortality, valve re-intervention
		Offer every patient heart rehabilitation program	Quality of Life
		Raise more attention to diet of the patient, practice spirometry	Quality of Life
		Introduce a checklist for the exit consult	Re-intervention
	Monitoring/ Managing	Adjust the medication protocol	Quality of Life
TAVR	Monitoring and preventing	Optimize Frailty identification	None*
		Introduce home monitoring system for measuring blood pressure (E-Health)	Quality of Life
	Diagnosis	Introduce more frequent TAVR team meetings to discuss patients	Mortality, Quality of Life
		Improve hospital logistics (with the support of the Lean method)	Mortality, Quality of Life
		Assure that an echo is always available before diagnosis	Complications
		More frequent TAVR Team meetings to discuss patients	Mortality
		Digitalize the treatment plan	Mortality
		Involve an anesthetist in the TAVR Team meetings	Mortality
		Introduce a diagnosis checklist for treatment choices	None*
	Preparing	Conduct pre-operative check-up and CT-scan on the same day	Waiting-times
		Introduce a checklist for the check-up	Mortality
		Involve an anesthetist much more this phase	Complications
		More local anesthesia	Mortality
		More procedures in one day or another day for TAVI procedure to shorten the waiting times	None*
	Intervening	Introduce the presence of a surgeon, cardiologist and anesthetist during the procedure	Complications
		Use ACIST Pump^c^ (control of injection rate)	None*
		Only use the new generation of valves (replaceable valves)	Mortality
		Use of a debris catch device	Stroke
	Recovery/Rehab	Introduce clinical pathway	Quality of Life
		Ensure removal of the pacemaker the following day and directly implant the long-term pacemaker if needed	Infections
		Apply telemetry monitoring for full period until dismissal	None*
	Monitoring/Managing	Define targets for medication	Re-intervention

*The proposed potential improvement intervention is not expected to have considerable impact on one of the patient-relevant outcome measures, but process or structure measures

^a^MECC is minimal extracorporeal circulation

^b^ICU is intensive care unit

^c^ACIST Pump simplifies contract injection for procedures

Step 4 resulted in an overview of five local initiatives that were implemented in the period of the first research step (Table 3). We ordered the improvement interventions according to treatment group (SAVR or TAVR). The identified intervention, with an impact on both long-term survival and 30-day mortality, measured a frailty score before hospitalization for TAVR. Frailty is part of the MB measures as an initial condition.

B: Selection of an improvement intervention

- Step 1: Causal chains and intermediate outcomes

**Table 3:** Monitoring overview

MONITORING IMPROVEMENT								
Treatment	Indicator	Initiative	Based on outcome measures yes/no	How did it take place?	Implementation date	Intended impact on which outcome	Implementation completion (%)	How is it measured?
TAVR	30-day mortality	1) Pre-TAVR/frailty outpatient clinic started in 2014, 2) TAVR complication discussion started in 4th quarter 2014 with the following issues discussed: A) Choice of valve selection, B) Creation of a specialization team, C) Add additional CT images in report to the TAVI Team.	yes		1st quarter 2015	30-day mortality	100%	Valve choice: registry measured
	1-year mortality	Pre-TAVR/frailty outpatient clinic started in 2014	yes		4th quarter 2014	1-year mortality	100%	Not
	long-term survival	Proposal change training plan - development of online course small private online course for residents with focus on frailty, functional decline and shared decision making	no	specific project team for elderly care	4th quarter 2015	none	0%	Not
	Vascular complications	1) Routine CT scan required pre-TAVR, 2) Start study new closing device in 2015, 3) Start complication discussions in 4th quarter 2014, where it was discussed to lower the threshold for a surgical cut down	yes		4th quarter 2014	Vascular complications	100%	Not
SAVR	Re-sternotomy	Coagulation policy: Optimization of the transfusion policy based on for example the TEG^a^ at the operation room, or no coagulation correction. In addition, the aim is to reduce the number of blood transfusions. The number of re-sternotomies could decrease at a targeted corrected clotting status of the patient.	no	Initiative from Anesthesiologists who conducted research	1st quarter 2015	Bleeding complications	50%	As part of a study

^a^TEG thromboelastography for testing the efficiency of blood coagulation

Causal chains were constructed for each improvement intervention resulting in eighteen causal chains for SAVR and twenty-two for TAVR.

For SAVR we ranked three causal chains with three stars for the impact on outcome measures, specifically long-term survival. These initiatives were: implementing an anticoagulation policy, offering a cardiac rehabilitation program to all patients, improving preoperative nutritional status of patients and paying more attention to the frail and elderly. For TAVR, we ranked four causal chains with three out of three stars for impact on patient-relevant outcome measures: improve speed of treatment decision, determine a frailty score in the prevention phase, introduce a checklist for the preoperative check-up and improve logistics with the Lean methodology. Two interventions presented no impact on patient-relevant outcome measures, but rather on cost savings. These were, firstly, develop a clinical pathway for the recovery phase, and, secondly, carry out echocardiography only on indication.

- Step 2: Consensus decision

We presented the results to the multidisciplinary team, who, through discussion, took a consensus decision on potential improvement interventions with the highest impact on outcome measures from phase A. The adjusted Delphi method resulted in a top four improvement intervention overview for both treatments, which was further discussed in the multidisciplinary team. The multidisciplinary team was specifically interested in an initiative that would change the treatment plan and the process of both treatments, because of the expected highest impact on outcomes. Also, as the aim was to select only one final improvement initiative, the impact on patient-relevant outcomes would be bigger with an initiative that suited both the SAVR and TAVR treatment. Since interventions targeting the frail elderly were mentioned most frequently in the multidisciplinary team and the older age category was associated with 30-day mortality, we decided to focus on more attention to the diet of our patients. The decision was taken with a specific intervention plan to improve the nutritional status and condition of older patients through a protein-enriched diet before the operation. We opted for this initiative because of its potential impact on long-term survival, 30-day mortality and also a cost measure, namely length of stay.

### A toolbox for the identification and selection of an improvement intervention

**Fig. 5:**
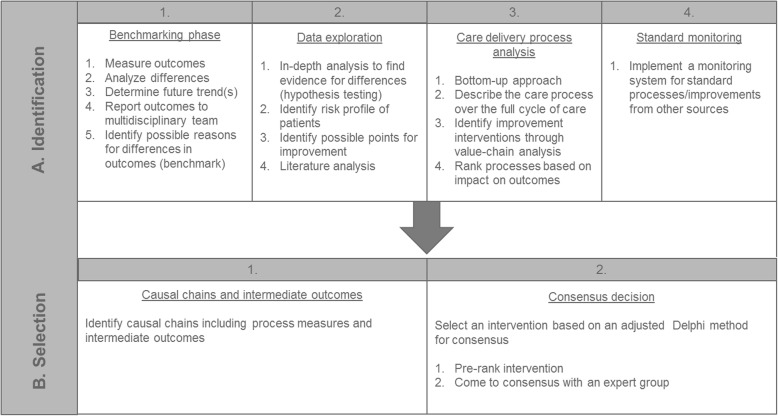
The Intervention Selection Toolbox (IST). The IST presents steps for two phases for identifying and selection improvement interventions based on patient-relevant outcome measures

On the basis of existing quality improvement (QI) programs and our experiences from the process we developed an integrated and combined approach from both patient-relevant outcomes and processes to identify and select improvement interventions aiming at improving quality of care: the Intervention Selection Toolbox (IST) (Fig. 5). The IST was tested and applied to improve the quality of care for aortic valve disease. IST consists of two phases to identify improvement interventions with an expected high impact on outcome measures. In phase A: Identification, the following steps were identified: 1. Benchmarking, 2. Data exploration, 3. Care delivery process analysis and 4. Standard monitoring. In phase B: Selection, two steps were identified: 1. Causal chains and intermediate outcomes and 2. Consensus decision. The steps of the IST are generically described in Additional file 4.

## Discussion

### Meaning of findings

This paper delivered a toolbox for identifying and selecting improvement interventions, the IST, as well as the selection of an improvement intervention for the treatment of aortic valve disease in the primary hospital of investigation.

We developed the identification and selection toolbox based on existing methods from the literature [5, 11, 14, 15]. The challenges with designing complex interventions have earlier been described [25]. The IST is unique, as its focus is on the design of an improvement intervention with the highest expected impact on outcomes for patients instead of processes, but it does not neglect processes. For the IST, outcomes and processes are combined into one toolbox. Earlier frameworks focus on the optimization of interventions [25]. Whereas, the IST focusses on the identification of improvement potential for outcomes by identifying and selecting an improvement intervention. As Donabedian stressed, only by connecting structure, process and outcome quality improvement can be achieved [26]. This is often forgotten in other improvement models. VBHC was introduced with the promise to solve the cost crisis [27]. But, how outcome measures should be used for improving quality of care and reducing costs, was not described. Measurement forms the basis for improvements in health care. With the help of these measurements, a feedback loop on what is the current state of health care can be implemented. As suggested by the VBHC concept, outcome measures are needed to introduce competition to tempt professionals to improve care for patients [28]. In order to find adequate QI interventions it is not sufficient to merely measure and benchmark outcome measures. Additional data analysis and process analysis will lead to new ideas that will have the potential to improve beyond best practices from benchmarking. The IST combines the strength of both strategies: 1) to analyze and compare health outcomes and 2) to analyze and study the care delivery process and find clues for improvement. Most approaches so far focus on one of both strategies.

The overall goal is to achieve statistically significant and clinically relevant improvements in patient-relevant outcomes. To determine these statistically significant improvements in patient-relevant outcomes, we often need long follow-up periods and big samples. In order to achieve this goal we could use the intermediate outcomes that give insights into improvements on a smaller scale to predict an effect on patient-relevant outcomes.

To ensure a successful identification and selection of improvement interventions certain barriers and facilitators have to be considered. Barriers and facilitators could be relevant on the following levels: (1) the readiness to change of individual care providers, (2) social context, (3) organizational context, and (4) economic and legal context [5]. Skills, attitude, resources, and regulations could hinder a successful improvement toolbox implementation [5]. In order to facilitate a successful implementation, a preliminary context and resource analysis could strengthen the success of the toolbox. If the multidisciplinary team was not ready for improvement, the results and overall success of this investigation would certainly have been different. Moreover, the selection of an intervention is influenced by its feasibility. An improvement intervention that was not feasible for implementation was more easily disregarded by the multidisciplinary team. It is, thus, important that the above-mentioned barriers are firstly identified to prevent unsuccessful processes.

Improvement interventions that were identified, but not selected need to remain under the attention of the multidisciplinary team. We presented the interventions identified in our study to the multidisciplinary team for further decision making. Further implementation could follow from the pool of identified interventions if required.

### Limitations

Our study has some limitations that need to be mentioned. The hospital of investigation had a general aim of improving patient-relevant outcomes in the strategic plan. Hence, the ambition of the multidisciplinary team might be driven by the overall movement toward improvement. In order to fully evaluate this approach, it would need to be tested in several different settings and for different medical conditions for transferability. The proof of principle of the IST will come from analyzing the impact of the resulting improvement initiatives in practice. The protein-enriched diet for preoperative optimization will be implemented and evaluated within the primary hospital.

The starting point for identifying and selecting improvement interventions is the availability of outcome data. In the current situation, the IST was applied by using available local outcome data which was part of a Dutch clinical outcome registry [20]. The use of local data might have affected the results of the current study. In order to apply the IST an outcome registry accelerates the identification and selection process.

Following the steps of the IST offered valuable insights into improvement of care processes based on outcomes. However, in our case it was relatively time-consuming to follow all the steps for care professionals, considering the amount of multidisciplinary team meetings and analyses to be conducted. In further research it should also be tested whether the phases and steps could be followed quicker. For this study, we did not evaluate how experts have experienced this process. On the other hand, it has not yet been evaluated what the results would have been if another approach was chosen. When a different sequence of the steps was opted for, the results could possibly have been different. Also, if certain steps would not have been taken or additional steps had been added to the toolbox, the results might have changed. To minimize these possibilities of different results, an evaluation should be conducted in future studies. Furthermore, in our approach one improvement intervention was selected to suit two treatments of aortic valve disease. This made the decision for one suitable intervention more complex. Further research applying the toolbox could test whether choosing one improvement per treatment would lead to better results. The toolbox development is based on a case study and not an evidence-based improvement or clinical trial. Moreover, further validation in another case is required in order to test transferability.

## Conclusion

The IST combines care delivery process analyses and outcome analyses and offers a practical guide on how to identify and select improvement interventions based on VBHC. The approach identified within this study could guide other hospitals in the selection of high-impact improvement interventions.

## Supplementary information

**Additional file 1.** Definitions of variables and coding (21).

**Additional file 2.** CDVC ranking list example.

**Additional file 3.** Example evaluation tool from CDVC.

**Additional file 4.** Generic description of the steps the IST.

## Data Availability

The datasets used and analysed during the current study are available from the corresponding author on reasonable request.

## Abbreviations

VBHC: Value-based health care
MB: Measurably better
SAVR: Surgical aortic valve replacement
TAVR: Transcatheter aortic valve replacement
QI: Quality improvement
