# Supplementary material for: Selecting interventions to improve patient-relevant outcomes in health care for aortic valve disease – the Intervention Selection Toolbox
Source: BMC Health Serv Res. 2020 Mar 19;20:232. doi: 10.1186/s12913-020-05090-z (PMC7082899; doi:10.1186/s12913-020-05090-z)
Supplement: Supplementary file 1 — Additional file 1. Definitions of variables and coding (21). [file 12913_2020_5090_MOESM1_ESM.docx]

**Additional file 1** Definitions of variables and coding (21).

| **Name variable** | **Definition** | **Coding** |
| --- | --- | --- |
| **Outcome measures SAVR** | | |
| Long-term survival | Patients who survive as a result of the number of days elapsed after the intervention with a maximum follow-up of 5 years. | Not applicable |
| 120-day mortality | Patients who die regardless of cause of death within 120 days (≤ 120 days) after intervention. | 3 categories: 0=no mortality, 1=mortality, 9=unknown |
| 30- day mortality | Patients who die regardless of cause of death within 30 days (≤ 30 days) after intervention. Excluding mortality during the procedure (procedural mortality) (≤ 0 days). | 3 categories: 0=no mortality, 1=mortality, 9=unknown |
| Quality of Life | Quality of life of the patients measured before and after intervention. Measurement before intervention=measured no longer than a maximum of 2 months before intervention. Measurement after intervention=measured between 10-14 months after intervention. Measured with the Short Form (36) Health Survey. | Not applicable |
| Cerebrovascular accident (CVA) | Patients for which a neurological determination of a postoperative stroke has occurred within 72 hours (≤ 72 hours) after intervention (excluding Transient Ischemic Attack). | 3 categories: 0=no CVA, 1= CVA, 9=unknown |
| Deep sternal wound infection | Deep sternal wound infection developing within 30 days (≤ 30 days) after intervention. It is assumed that the patient returns to the treatment hospital. | 3 categories: 0=no deep sternal wound infection, 1=deep sternal wound infection, 9=unknown |
| Implantation of a new permanent pacemaker | Post-operative implantation of a new (no replacement) permanent pacemaker within 30 days (≤ 30 days) after intervention. | 3 categories: 0=no implantation new permanent pacemaker, 1= implantation new permanent pacemaker, 9=unknown |
| Freedom of valve-re-intervention | Patients who are free from aortic valve re-intervention (aortic valve replacement, aortic valve repair or percutaneous paravalvular leakage (PVL) closure) on the same aortic valve as a function of the number of days elapsed after intervention. | 3 categories: 0=no valve re-intervention, 1=valve re-intervention, 9=unknown |
| Initial conditions SAVR | | |
| EuroSCORE | Last determined logistic EuroSCORE II measured before the intervention (measured continuously in % with 2 decimals). | 3 categories: 1=low < 10%, 3=high > 10%, 9=unknown |
| Outcome measures TAVR |  |  |
| Long-term survival | Patients who survive as a result of the number of days elapsed after the intervention with a maximum follow-up of 5 years | Not applicable |
| 30-day mortality | Patients who die regardless of cause of death within 30 days (≤ 30 days) after intervention. | 3 categories: 0=no mortality, 1=mortality, 9=unknown |
| Quality of Life | Quality of life of the patients measured before and after intervention. Measurement before intervention=measured no longer than a maximum of 2 months before intervention. Measurement after intervention=measured between 10-14 months after intervention. Measured with the Short Form (36) Health Survey. | Not applicable |
| Cerebrovascular Accident (CVA) | Patients for which a neurological determination of a postoperative stroke has occurred within 72 hours (≤ 72 hours) after intervention (excluding Transient Ischemic Attack). | 3 categories: 0=no CVA, 1= CVA, 9=unknown |
| Implantation of a new permanent pacemaker | Post-operative implantation of a new (no replacement) permanent pacemaker within 30 days (≤ 30 days) after intervention. | 3 categories: 0=no implantation new permanent pacemaker, 1= implantation new permanent pacemaker, 9=unknown |
| Vascular complications | Patients who develop a vascular complication within 30 days (≤ 30 days) (diagnosis according to the VARC-2 definition) from the start of the intervention (including pre-operative vascular complications). | 3 categories: 0=no vascular complications, 1=vascular complications, 9=unknown |
| Freedom of valve re-intervention | Patients who are free from aortic valve re-intervention (aortic valve replacement, aortic valve repair or percutaneous paravalvular leakage (PVL) closure) on the same aortic valve as a function of the number of days elapsed after intervention. | 3 categories: 0=no valve re-intervention, 1=valve re-intervention, 9=unknown |
| Explanatory variables TAVR | | |
| Access route | Applied access route for TAVR. | 4 categories: 1=transfemoral , 2=transapical and subclavian , 4=direct aortic and transaxillary, 9=unknown |
| Previous heart operation | Patients who have undergone previous cardiac surgery, with opening of the pericardium, prior to the intervention throughout life. | 3 categories: 0=no previous heart operation, 1=previous heart operation, 9=unknown |
| Previous stroke | Patients who have undergone a CVA prior to the intervention throughout life (excluding TIA). | 3 categories: 0=no previous CVA, 1=previous CVA, 9=unknown |
| Previous mitral valve stenosis | Patients with mitral valve stenosis that has been diagnosed prior to the intervention. Measured with the last echocardiogram up to 1 year before intervention. | 3 categories: 0=no or mild mitral valve stenosis, 1=moderate/serious mitral valve stenosis, 9=unknown |
| Hospital | Measurably Better hospitals participating in the study that offered data from 2010-2014. | 100=Primary hospital, 3=B, 4=C, 6=D, 8=E |
| Urgency of the procedure | Urgency of the procedure. Elective means that patients have a routine intake for the intervention. Urgent means patients who are not elective for the operation but need an intervention within the current admission for medical reasons. These patients cannot be sent home without a definitive procedure. Emergency and rescue cases were excluded. | 3 categories: 1=elective, 2=urgent, 9=unknown |
| Severe left ventricular dysfunction | Left ventricular dysfunction is expressed as an ejection fraction (EF, in %). The registered EF may not have been established for more than 6 months prior to intervention. The last measured EF before intervention is used. | 4 categories: 1=EF>50%, 2=EF 30-50%, 3=EF<30%, 9=unknown |
| Age | Age in years at start of the intervention. | Not applicable |
| Renal dysfunction | Renal dysfunction is calculated based on the creatinine level and is defined as a reduced glomerular filtration rate (GFR) of <60 ml/min/1.73 m2. The GFR is calculated according to the MDRD formula. | 3 categories: 0=no renal dysfunction, 1=renal dysfunction, 9=unknown |
