# Supplementary material for: Selecting interventions to improve patient-relevant outcomes in health care for aortic valve disease – the Intervention Selection Toolbox
Source: BMC Health Serv Res. 2020 Mar 19;20:232. doi: 10.1186/s12913-020-05090-z (PMC7082899; doi:10.1186/s12913-020-05090-z)
Supplement: Supplementary file 2 — Additional file 2. CDVC ranking list example. [file 12913_2020_5090_MOESM2_ESM.docx]

**Additional file 2** CDVC ranking list example

|  | | | | |
| --- | --- | --- | --- | --- |
|  | **1. IMPACT on outcomes** | **2. ROOM for improvement** | **3.**  **FEASIBILITY to improve** | **Comment** |
| Monitoring and preventing |  |  |  |  |
| preventive measures within hospital |  |  |  |  |
| Diagnosing |  |  |  |  |
| Waiting times |  |  |  |  |
| Assessment results of imaging |  |  |  |  |
| Anamnesis |  |  |  |  |
| Defining treatment plan |  |  |  |  |
| Preparing |  |  |  |  |
| pre-operative policlinic |  |  |  |  |
| Pre-operative check-up |  |  |  |  |
| Intervening |  |  |  |  |
| Access route |  |  |  |  |
| Access route closure |  |  |  |  |
| Volume (number of procedures) |  |  |  |  |
| Recovering/Rehab |  |  |  |  |
| In hospital recovering |  |  |  |  |
| Regular checkups |  |  |  |  |
| Support |  |  |  |  |
| Counselling/education on prevention |  |  |  |  |
| Monitoring/Managing |  |  |  |  |
