# Supplementary material for: Selecting interventions to improve patient-relevant outcomes in health care for aortic valve disease – the Intervention Selection Toolbox
Source: BMC Health Serv Res. 2020 Mar 19;20:232. doi: 10.1186/s12913-020-05090-z (PMC7082899; doi:10.1186/s12913-020-05090-z)
Supplement: Supplementary file 3 — Additional file 3. Example evaluation tool from CDVC. [file 12913_2020_5090_MOESM3_ESM.docx]

**Additional file 3** Example evaluation tool from CDVC

| **Improvement possible?** | | | **Yes** | | **No** | |
| --- | --- | --- | --- | --- | --- | --- |
|  |  |  |  | |  | |
| **No.** | **Potential improvement intervention** | **Influences which outcome** | **Follow-up action** | **Comment** | | **Who** |
| 1 | ... | ... | ... | ... | | ... |
| 2 | … | ... | … | ... | | ... |
| 3 | … | ... | … | ... | | ... |
