# Supplementary material for: Selecting interventions to improve patient-relevant outcomes in health care for aortic valve disease – the Intervention Selection Toolbox
Source: BMC Health Serv Res. 2020 Mar 19;20:232. doi: 10.1186/s12913-020-05090-z (PMC7082899; doi:10.1186/s12913-020-05090-z)
Supplement: Supplementary file 4 — Additional file 4. Generic description of the steps the IST. [file 12913_2020_5090_MOESM4_ESM.docx]

Additional file 4. Generic description of the steps the IST

1. Identification

The identification of potential for improvement consists of four steps in order to establish a number of interventions that are expected to contribute to improved outcomes.

- 1. Benchmarking: Benchmarking includes: the identification of outcomes to be benchmarked, organizations to benchmark with, data collection, analysis of differences, determination of future trend and sharing results with a multidisciplinary team of experts. The multidisciplinary team comments and formulates hypotheses for the explanation of identified differences.
  2. Data exploration: Data exploration aims at understanding data and their characteristics. In this step, the formulated hypotheses of the previous steps are tested with the help of statistical hypothesis testing. Possible risk groups or risk factors are identified. Next to statistical analyses, literature study may support hypothesis testing.
  3. Care delivery process analysis: The care process(es) need to be described in detail. The care delivery value chain developed by Porter et al (2008) may support this process. Important is to consider the processes of the full cycle of care. After description, a prioritization by a multidisciplinary team follows, who rank the processes based on its potential impact on outcomes and feasibility to change.
  4. Standard monitoring: In order to identify interventions with highest expected impact on outcomes, a list describing all improvement interventions with impact on outcomes needs to be established. The aim of the list is not to eliminate ongoing improvement interventions, but rather to get an overview of improvement interventions that are aimed to improve the same outcomes under investigation. The standard monitoring can be a dashboard or simply a list of improvement interventions.

1. Selection

The selection of an improvement intervention consists of two steps aiming to choose the improvement intervention with highest expected impact on outcomes.

- 1. Causal chains and intermediate outcomes: The causal relation between a potential improvement intervention and the outcome measures, including process measures and intermediate outcome measures needs to be described. This can be used to estimate the potential impact of the improvement intervention on outcomes.
  2. Consensus decision: Based on expert opinions of a multidisciplinary team, consensus on one (or more) intervention(s) needs to be taken. By ranking the interventions, a multidisciplinary team of experts can choose the intervention(s) with highest expected impact on outcomes.
